# How did the amphibious *Eleocharis vivipara* acquire its C_3_–C_4_ photosynthetic plasticity?

**DOI:** 10.1111/jipb.13813

**Published:** 2024-11-27

**Authors:** Guillaume Besnard

**Affiliations:** ^1^ CNRS, Université Paul Sabatier, IRD, INP Toulouse, UMR 5300 CRBE (Centre de Recherche sur la biodiversité et l'Environnement) Toulouse 31062 France

The recently published study by Liu et al. (2024) on a high‐quality, chromosome‐level genome of *Eleocharis vivipara* provides new insight into the multiple evolution of C_4_ photosynthesis in Cyperaceae and in particular in *Eleocharis*. The species studied has the rare feature of alternately using C_3_ photosynthesis underwater and C_4_ photosynthesis on land (Ueno et al., 1988), making it an exciting model to better understand the genetic control and evolution of the C_4_ trait and, in particular, the evolutionary challenge to switch from C_3_ to C_4_ photosynthesis from the aquatic to the terrestrial environment. This may imply both the control of genes involved in the C_4_ pathway and deep cellular anatomical changes. Alternately using C_3_ or C_4_ photosynthesis may also lead to evolutionary trade‐offs (e.g., optimization of photosynthetic enzymes in contrasting C_3_ and C_4_ biochemical contexts). Maintaining C_3_ and C_4_ genes may therefore be necessary; hybridization (e.g., allopolyploidization) between non‐C_4_ and C_4_ taxa could have been involved to favor the emergence of such facultative photosynthetic strategy.

Wide variation in photosynthetic type has been previously reported within *Eleocharis* (Murphy et al., 2007). Phylogenetics of this genus have supported the idea that C_4_ photosynthesis has been derived at least three times, with several cases of possible reversion to C_3_‐like or intermediate pathways and several additional origins of C_3_–C_4_ intermediate photosynthetic pathways (Roalson et al., 2010). Inferring such transitions based solely on species phylogenies, however, can be tricky (Christin et al., 2010), requiring consideration of other evidence. In another study, genes encoding C_4_ PEPCs were investigated in two phylogenetically distant C_4_ species, *Eleocharis baldwinii* and *E. vivipara* (Besnard et al., 2009). Unexpectedly, C_4_ PEPC genes have recently diverged between the two *Eleocharis* species, supporting the idea that one of them has borrowed this core C_4_ gene from the other lineage by hybridization or by a horizontal transfer event, as widely reported in grasses (Bianconi et al., 2020). As *E. vivipara* belongs to a monotypic C_4_ lineage, the C_4_ PEPC genes were probably obtained from the more diverse *E. baldwinii* clade (Roalson et al., 2010; Larridon et al., 2021).

In their study, Liu et al. (2024) used genomic and transcriptomic data to document karyotype evolution within the Cyperaceae family and the origin of C_4_ genes in *E. vivipara*, and then to achieve new insight into the genetic control of C_3_ and C_4_ photosynthesis in this amphibious species. This group first demonstrated that holocentromeric chromosomes in *Eleocharis* should favor genomic reorganizations and, in turn, hybrid speciation. This could have promoted gene exchange between distantly related lineages. They also demonstrated that, although *E. vivipara* is tetraploid, C_4_ genes were evenly distributed in the two subgenomes A and B, strongly suggesting that C_4_ photosynthesis had predated the polyploidization event in its ancestor. Liu et al. then dated a whole genome duplication (WGD) at 3.5 Mya based on the divergence between the two subgenomes. However, allopolyploidy between two C_4_ taxa should be at the origin of the currently sequenced accession, meaning that the WGD could have been a more recent event than this estimate suggested. Finally, they demonstrated that epigenetic control allowed integrated responses to water deprivation during the C_3_ to C_4_ switch involving various biological processes related to photosynthesis and anatomy. This also confirmed that the transition from aquatic to terrestrial environment involved a huge reprogramming of gene expression.

While Liu et al. (2024) have brought very important insight into the understanding of the (epi)genetic determinants governing photosynthetic transition and chromosomal organizations favoring hybrid stability, some questions remain open, in particular on when and how did the ancestor(s) of *E. vivipara* acquire C_4_ photosynthetic traits. This is important to better understand the multiple evolution of the C_4_ trait within *Eleocharis*. Was the diploid ancestor of *E. vivipara* already a facultative C_3_ versus C_4_ species? When did the exchange of C_4_ genes between distantly related extent C_4_ *Eleocharis* lineages, as suggested by previous phylogenetic works, occur? How extensive were the genomic reorganizations following the WGD event detected by Liu et al., notably with regard to gene retention from each ancestral *Eleocharis* genome (especially for C_4_ genes)? Liu et al. provide new genomic and transcriptomic resources that will be extremely useful to address such questions with a phylogenomic approach (e.g., Dunning et al., 2019). By reconstructing gene phylogenies and comparing the genome location and the synteny of DNA fragments carrying C_4_ genes between different *Eleocharis* species, especially between *E. vivipara* and close relatives of *E. baldwinii*, it would now be possible to retrace the evolutionary history of core C_4_ genes within the genus, as well as to study the genomic reorganizations that are particularly frequent in this clade (Roalson, 2008; Liu et al., 2024). The work of Liu et al. thus represents an important step toward a better understanding of C_3_–C_4_ photosynthetic plasticity in an amphibious lineage. Such knowledge could have major application in the improvement of crops growing under various hydric conditions (e.g., rice), although consideration of the ecological consequences of such manipulations should still be measured.

## CONFLICTS OF INTEREST

The authors declare no conflict of interest.
